# Supplementary material for: PDE and agent based simulation approaches to Ischemic Dermal Wound Closure
Source: PLoS One. 2026 May 6;21(5):e0340624. doi: 10.1371/journal.pone.0340624 (PMC13148719; doi:10.1371/journal.pone.0340624)
Supplement: Attachment — Submitted filename: rebuttal_letter_avner_4.docx [file pone.0340624.s003.docx]

**To: The editors and reviewers of Plos One,**

Dear editors and reviewers,

We are pleased and privileged to resubmit our manuscript “PDE and Agent Based Simulation Approaches to Ischemic Dermal Wound Healing" for your reconsideration to be published in Ploe One.

We would like to thank the reviewers for their kind words and detailed review. We believe that after addressing the comments, the paper is much clearer and better structured for potential readers. In particular, we paid special attention to showing the model capture *in vivo* data.

To make the review process as smooth as possible, we highlight all the additions and edits in the manuscript in red and blue fonts. In addition, a formal cover letter detailing our responses to the reviewers’ comments point-by-point is attached below.

Kind regards,

The authors

**Reviewer #1:**

**Comment:** “The comparison with in vivo data (Ref. [55]) is superficial. It is limited to a qualitative visual inspection of figures, without any quantitative validation. No comparison method is described in the Methods, and the Results lack the rigor required for scientific validation. Moreover, the assignment of ischemic level α = 0.5 to represent the ischemic model in Ref. [55] is arbitrary. In addition, the re-epithelialization data are inconsistent: in Ref. [55], non-ischemic wounds show ~60% closure at Day 7, whereas the model predicts only ~20% (α = 0). Therefore, the model and the experimental data are not consistent.The authors emphasize that the two mathematical models (PDE and ABS) are in close agreement with each other. However, what matters is agreement with experimental reality, not merely internal consistency between two simplified models. Agreement between models that both deviate from in vivo data does not strengthen credibility. The limitation regarding the lack of mapping between the ischemic parameter α and physiological indices remains unresolved. Ref. [55] reports SPP values for each wound model, but since the present study’s predictions are already inconsistent with the experimental wound closure data (see comment#1), no meaningful mapping between α and clinical indices can be established. The origin of this discrepancy with in vivo results is not entirely clear. It may stem from parameter inaccuracies, or from oversimplified modeling assumptions—for example, the flat “2D epidermis” assumption (regarding the comment #3). Such simplifications may critically distort wound healing dynamics. While further refinement of the model may eventually address these issues, in its current form the model does not achieve biological plausibility. Because the manuscript’s central claim of experimental consistency is unsupported, and the models fail to reproduce established in vivo wound healing data, the study does not provide reliable or clinically translatable predictions. I therefore recommend rejection of this version.”

**Response:** Thank the reviewer for these comments, and we accordingly made changes in the manuscript in RED.

The reviewer is correct in, and we thank him for, criticising our sloppy assertion about the agreement between our model’s simulations and [55]. We have now performed correctly the simulation of wound closure, noting that wound closure in [55] Fig. 3 is given not by R(t) but by the percentage of R(t)/R(0) and that R(0) = 0.2 in [55]) (not R(0) = 1cm in our model). The simulation results now fit well with [55] Fig. 3 as seen in the new Fig. 4B.

Note that we started these simulations at day 3 since our model does not include the process of initial wound stretching, which takes place initially for several days (as noted in item (4) of the model limitation, page 20).

With respect to epithelialization in [55] Fig. 4 for days 2 and 7, we note that the results in [55] were derived by mason tricome stain over the entire wound tissue, while our results on keratincyte density (in Fig. 3) are taken in the region where R(t) < r < R(0). Furthermore, and more importantly, the initial stretching of the wound (which is not included in our model) significantly affects epithelialization. We therefore do not expect keratinocyte density in our model (with radius of percentage of R(t)/R(0), and with R(0) = 0.2cm) to fit the epithelialization in [55] Fig. 4; hence, this is not included in the present version.

**Reviewer #2:**

**Comment: “**Oxygen therapy for ischemic wounds has been under clinical research, but there are many doubts about how to evaluate its effectiveness.Sincerely appreciate the authors for making substantial revisions based on the reviewers' comments, clarifying many uncertainties, and significantly enhancing the discussion section with additional and improved content.”

**Response:** Thank you for the positive comment.

**Reviewer #4:**

**Comment: “**To further support your conclusions, please discuss HBOT vs TCOT results. Is the difference in efficacy of the two methods comparable to those of in vivo? Does your model generate some hypotheses that can be tested in vivo?”

**Response:** We added a new paragraph (in Blue) on page 16. TCOT is not used in wounds that are just ischemic. But we think it is interesting to see what benefit it could potentially have compared to TOT.
