# Supplementary material for: PDE and agent based simulation approaches to Ischemic Dermal Wound Closure
Source: PLoS One. 2026 May 6;21(5):e0340624. doi: 10.1371/journal.pone.0340624 (PMC13148719; doi:10.1371/journal.pone.0340624)
Supplement: Attachment — Submitted filename: rebuttal_letter_avner_4_minor.docx [file pone.0340624.s004.docx]

**To: The editors and reviewers of Plos One,**

Dear editors and reviewers,

We are pleased and privileged to resubmit our manuscript “PDE and Agent Based Simulation Approaches to Ischemic Dermal Wound Healing" for your reconsideration to be published in Ploe One.

We would like to thank the reviewer for his/her positive review and suggestions on how to improve the structure of the manuscript. To this end, we highlight all the additions and edits in the manuscript in red font. In addition, a formal cover letter detailing our responses to the reviewers’ comments point-by-point is attached below.

Kind regards,

The authors

**Reviewer #1:**

**Comment:** “The comparison between the present model and the in vivo data reported in Ref. 55 is now more convincing than in the previous version. The agreement demonstrated in Fig. 4 appears reasonable and provides a certain level of support for the model. However, the methodology used for this comparison is not described in the Methods section. Instead, several methodological details appear only within the Results section, where Methods and Results are intermixed. Once the comparison methodology is appropriately moved and clarified, I believe the manuscript will meet the standard for publication..”

**Response:** Thank you for this comment. We added at the beginning of the results section an outline of the computation used to clarify this point.
